# Spatial Patterns of *Thalassia testudinum* Immune Status and *Labyrinthula* spp. Load Implicate Environmental Quality and History as Modulators of Defense Strategies and Wasting Disease in Florida Bay, United States

**DOI:** 10.3389/fpls.2021.612947

**Published:** 2021-02-05

**Authors:** Paige Duffin, Daniel L. Martin, Bradley T. Furman, Cliff Ross

**Affiliations:** ^1^Department of Biology, University of North Florida, Jacksonville, FL, United States; ^2^Department of Genetics, University of Georgia, Athens, GA, United States; ^3^Florida Fish and Wildlife Research Institute, Florida Fish and Wildlife Conservation Commission, St. Petersburg, FL, United States

**Keywords:** immunocompetence, host-pathogen interactions, hyposalinity stress, opportunistic pathogens, environmental fluctuation, anthropogenic influences, resistance, tolerance

## Abstract

Seagrass wasting disease, caused by protists of the genus *Labyrinthula*, is an important stressor of the dominant macrophyte in Florida Bay (FB), United States, *Thalassia testudinum*. FB exhibits countervailing gradients in plant morphology and resource availability. A synoptic picture of the *Thalassia-Labyrinthula* relationship was obtained by assessing the activity of four immune biomarkers in conjunction with pathogen prevalence and load [*via* quantitative PCR (qPCR)] at 15 sites across FB. We found downregulated immune status paired with moderate pathogen load among larger-bodied host phenotypes in western FB and upregulated immunity for smaller-bodied phenotypes in eastern FB. Among the highest immune response sites, a distinct inshore-offshore loading pattern was observed, where coastal basins exposed to freshwater runoff and riverine inputs had the highest pathogen loads, while adjacent offshore locations had the lowest. To explain this, we propose a simple, conceptual model that defines a framework for testable hypotheses based on recent advances in resistance-tolerance theory. We suggest that resource availability has the potential to drive not only plant size, but also tolerance to pathogen load by reducing investment in immunity. Where resources are more scarce, plants may adopt a resistance strategy, upregulating immunity; however, when physiologically challenged, this strategy appears to fail, resulting in high pathogen load. While evidence remains correlative, we argue that hyposalinity stress, at one or more temporal scales, may represent one of many potential drivers of disease dynamics in FB. Together, these data highlight the complexity of the wasting disease pathosystem and raise questions about how climate change and ongoing Everglades restoration might impact this foundational seagrass species.

## Introduction

Seagrass meadows form the benthic habitat of some of the most globally significant and ecologically diverse communities in the aquatic realm (Robblee et al., 1991; Zieman et al., 1999; Orth et al., 2006). As autogenic and allogenic engineers, seagrasses are foundation species whose meadows provide a broad array of ecosystem services, including erosion control, carbon and nutrient cycling, and water column, as well as sediment oxygenation (Costanza et al., 1997; Waycott et al., 2009; Duarte et al., 2010). They also contribute directly and indirectly to secondary production that supports important commercial and recreational fisheries and ecotourism (Kitting et al., 1984; Heck et al., 1995). However, high light requirements, shallow coastal distributions, and proximity to human development make seagrasses especially vulnerable to ecological perturbation (Onuf, 1996; Ralph et al., 2007; Biber et al., 2009), so much so that they often serve as bio-indicators of ecosystem function and resilience (Hackney and Durako, 2004).

As the impacts of climate change intensify, predictions can be made regarding the persistence of seagrass-dominated communities across time and space (Short and Neckles, 1999). For example, growing evidence suggests that increased environmental fluctuation and recurrence of extreme weather events (i.e., marine heatwaves and altered precipitation; Donat et al., 2013) will negatively impact seagrasses through degraded water quality and physiological constraints (Waycott et al., 2009; Fraser et al., 2014; Peñalver et al., 2020). Another prediction is that climatic changes will lead to pathogen emergence, increased pathogen virulence, and/or the spread of pathogens to naïve host populations (Harvell et al., 1999; Burge et al., 2014; Cohen et al., 2018). This poses a significant mass mortality risk to densely packed seagrass monocultures (Waycott et al., 2009), similar to trends already detected in terrestrial systems (Fey et al., 2015). Further, the well-documented histories of environmentally- (Robblee et al., 1991; Hall et al., 2016) and disease-driven die-offs (Tutin, 1938; Short et al., 1987) suggest that understanding host-pathogen dynamics will be critical for managing seagrass resilience in the decades to come.

Protistan stramenopiles of the genus *Labyrinthula* include a set of opportunistic pathogens that infect seagrasses worldwide with a lesion-forming “seagrass wasting disease” (SWD) that reduces productivity (Durako and Kuss, 1994) and contributes toward host mortality (e.g., Vergeer and den Hartog, 1994; McKone and Tanner, 2009; Bull et al., 2012; Martin et al., 2016; see Sullivan et al., 2013, 2018 for comprehensive literature review). In the tropical and sub-tropical Caribbean and Gulf of Mexico, *Labyrinthula* spp. cause wasting disease in the long-lived, climax seagrass species, *Thalassia testudinum* (turtlegrass; Robblee et al., 1991; Blakesley et al., 2002; Trevathan-Tackett et al., 2013). Recent manipulative work has shown that parasite virulence is a complex function of host competency, physiological stress, and immune status (Brakel et al., 2014, 2019; Trevathan-Tackett et al., 2015; Bishop et al., 2017; Duffin et al., 2020), making the *Thalassia-Labyrinthula* pathosystem an attractive model for testing host-parasite interactions across a range of environmental scenarios. Using newly-developed quantitative PCR (qPCR) and immunoassay techniques (Duffin et al., 2020), we examined the relationship between the immune status of *T. testudinum* and *Labyrinthula* spp. loading along well-documented resource (i.e., phosphorus and sediment depth), morphological (i.e., leaf area and above-ground biomass), and stress (i.e., salinity regime) gradients in Florida Bay (FB), Florida, United States.

Florida Bay is a semi-enclosed estuary bordered by the Everglades and Florida Keys, opening westward into the Gulf of Mexico and eastward through a series of cuts into the Atlantic Ocean (Schomer and Drew, 1982; Hall et al., 2006; Bricker et al., 2011; Herbert et al., 2011). Within the bay, a network of shallow carbonate mud-banks limits wind and tidal currents to create idiosyncratic basins at a variety of spatiotemporal scales (Nuttle et al., 2000; Rudnick et al., 2005; Hall et al., 2006; Lee et al., 2006). However, broad northeast-to-southwest gradients in tidal range, sediment depth, and phosphorus availability – a function of sediment availability and proximity to the Gulf of Mexico (Rudnick et al., 1999; Herbert and Fourqurean, 2009) – oppose a prominent southwest-to-northeast gradient in freshwater delivery (Boyer et al., 1997; Peterson and Fourqurean, 2001). Perpendicular to the alongshore gradients is the rapidly attenuated influence of runoff from the Florida mainland (McIvor et al., 1994; Nuttle et al., 2000).

The resultant salinity regime reflects a long history of upstream changes to the Everglades watershed, resulting in altered freshwater, nutrient, and carbon flows into FB (Hunt and Nuttle, 2007; Kelble et al., 2007; Stabenau and Kotun, 2012). Reductions in freshwater input have led to a contraction of estuarine conditions and associated biota (Tabb et al., 1962; Schmidt and Davis, 1978; Marshall et al., 2014), a process termed the “marinization” of FB that has driven increases in the distribution and dominance of *T. testudinum* monocultures, even within coastal basins.

Today, FB supports some of the most extensive seagrass meadows in the world (Fourqurean and Robblee, 1999; Hackney and Durako, 2004; Madden et al., 2009; Bricker et al., 2011), including *Thalassia testudinum*, *Halodule wrightii*, and *Syringodium filiforme* (Hall et al., 2006; Cole, 2017). *T. testudinum* is by far the dominant canopy-forming species, occupying some 2000 km^2^ of the bay’s 2,100 km^2^ (Zieman et al., 1989). Across FB, it responds to resource and stress gradients with remarkable phenotypic plasticity in above-ground biomass, canopy height, and C:P ratios (Frankovich and Fourqurean, 1997; Bricker et al., 2011). During the last half-century, large, dense *T. testudinum* meadows in west-central FB have undergone several mass mortality events (e.g., 1974: Schmidt and Luther, 2002; 1987: Robblee et al., 1991; and 2015: Hall et al., 2016), each driven by a combination of high bottom-water temperature, hypersalinity, and stratification leading to widespread anoxia, photosynthetic stress, and sulfide toxicity (Carlson et al., 1994; Hall et al., 1999; Zieman et al., 1999; Borum et al., 2005; Koch et al., 2007; Johnson et al., 2018). Also, during this time, varied wet/dry season intensity and tropical weather systems have resulted in fluctuating *T. testudinum* densities in the coastal basins due to hyposalinity and light limitation (Wilson et al., 2020).

*Labyrinthula* infection is thought to be widespread in FB and likely factors into lost productivity and mortality that follow periodic reductions in light availability (e.g., sediment resuspension events and algal bloom) and other physiological challenges presented to FB seagrasses (e.g., Robblee et al., 1991; Carlson et al., 1994; Durako and Kuss, 1994; Blakesley et al., 2002; Hall et al., 2016). Such challenges allow opportunistic pathogens, such as *Labyrinthula* spp., to capitalize on compromised host immunity (Bishop et al., 2017; Duffin et al., 2020). Given current climate predictions of the region (e.g., increased temperatures, altered precipitation, more frequent hurricanes, and sea level rise; Pederson et al., 2012; Koch et al., 2015; Carlson et al., 2018), and the ongoing restoration of the greater Everglades ecosystem (i.e., the Comprehensive Everglades Restoration Plan, CERP; Everglades Forever Act, 1994; Florida Forever Act, 2000; Water Resources Development Act, 2000) aimed at bringing increased freshwater delivery to the upper portions of the FB, it is likely that *Thalassia-Labyrinthula* interactions will play an important role in the resilience of *T. testudinum*-dominated meadows.

As an initial step toward understanding the dynamics of this complex pathosystem, we collected baseline data regarding host resistance (immune status) and *Labyrinthula* spp. load across the well-documented ecophysiographic landscape of FB (Zieman et al., 1989; Boyer et al., 1999; Herbert et al., 2011; Briceño et al., 2013). Our collections at the end of May 2015 coincided with the late dry season, during a period of advancing hypersalinity that is characteristic of FB in early summer (Kelble et al., 2007). However, long-term monitoring stations in the northeast region of the bay detected a brief freshwater pulse in the weeks leading up to our sampling. The timing of this event and coincidence with our synoptic survey captures a unique perspective of *Thalassia-Labyrinthula* associations in a natural system. Using antecedent environmental trajectories, host morphometric data, and spatial patterns in immuno-status and pathogen loading, we build a conceptual model of the *Thalassia-Labyrinthula* pathosystem rooted in prevailing theories of resistance and tolerance in phytopathology. While aspects of this model remain speculative, it provides a compelling framework to generate testable hypotheses for a wide variety of future explorations in this seagrass pathosystem.

## Materials and Methods

### Turtlegrass Sampling

A survey of 15 *T. testudinum* beds was conducted across FB in late May, 2015 (Supplementary Table S1), in tandem with the spring 2015 Fisheries Habitat Assessment Program (FHAP) survey of the region (see next section for details). Ten turtlegrass shoots were haphazardly collected (roughly 8–10 m apart) along each permanent FHAP transect site. Individual specimens were bagged, temporarily stored on ice, and processed on land no more than 4 h post-collection. For processing, the third rank blade of each sample was split longitudinally; one half was stored in −80°C for use in immune activity assays and the other was preserved in Drierite® (Sigma Aldrich, Darmstadt, Germany) desiccant for qPCR procedures.

### Long-Term Survey Data

#### Turtlegrass Morphometric Surveys

Turtlegrass abundance surveys were conducted every spring and fall (May and September/October, respectively), in coordination with the end of the dry and wet seasons of FB by FHAP, a component of the Monitoring and Assessment Plan (MAP) of Restoration, Coordination, and Verification (RECOVER) within the CERP (Everglades Forever Act, 1994; Florida Forever Act, 2000; Water Resources Development Act, 2000). Starting at the coordinates for each of the 15 sites included in this investigation (see Supplementary Table S1), a 50-m transect line was haphazardly subsampled 10 times within a 0.25-m^2^ quadrat for macrophyte cover-abundance data (using a modified Braun-Blanquet technique; see Fourqurean et al., 2003), shoot density, standing crop, epiphyte biomass, and leaf morphometrics for 10 individuals, chosen haphazardly across quadrats. We focused on leaf morphometric data (specifically mean total area, in cm^2^, per shoot) for the scope of our investigation as it is considered an important aspect of disease ecology (Burdon and Chilvers, 1982). Although these surveys have been conducted biannually since 2006, only the data collected between spring 2010 and spring 2015 were considered, as this time frame most directly reflects the conditions of the current standing crop of *T. testudinum*.

#### FB Salinity Monitoring Data

Salinity is often regarded as the most influential driver of environmental conditions in FB (Stabenau and Kotun, 2012), while also affecting *Labyrinthula* sp. *in vitro* and *in planta* dynamics (Martin et al., 2009; Bishop et al., 2017). We used two long-term water quality monitoring programs to characterize salinity trends at two temporal scales: monthly South Florida Water Management District (SFWMD) grab samples^1^ and daily mean summaries of Everglades National Park (ENP) continuous monitoring data [South Florida Natural Resources Center (SFNRC), n.d.].

The short-term scale featured daily salinity means over a 2-month period leading up to the 2015 sampling (April 1st to May 30th 2015). Ideally, all salinity measurements would be obtained from the SFWMD database, as the monitored regions coordinate perfectly with 13 of our 15 study sites (the remaining two, CAR and MAN, featured significant gaps in the SFWMD data). However, as this survey records only monthly values, we substituted in daily salinity data from the SFNRC DataForEver monitoring stations at locations matching 10 of our 15 sample sites to analyze the short-term temporal scale as follows: six of the ENP stations (BS, MK, JB, JK, LM, and MB) matched perfectly or were very close to our sampling locations (BLK, GAR, JOE, JON, LIL, and MAN, respectfully); another four sites (DK, LB, BK, and WB) were located close enough that we considered them a proxy for our sites (DUC, LON, RAN, and WHP; Supplementary Figure S1).

The long-term scale spanned ~4.5 years antecedent to our sampling point (January 2010 to May 2015), and was measured at roughly 1-month intervals. FHAP permanent transects are co-located with the SFWMD stations, so we relied on the SFWMD-DBHYDRO database to capture salinity (psu) measures at the permanent transect sites on a monthly basis, weather permitting. The protocol measures a suite of water quality parameters including salinity *in situ*, with a combination salinity-conductivity-temperature probe (Orion Model 140) 10 cm beneath the surface. Our investigation led us to consider whether more local and/or recent historical salinity conditions helped explain an intriguing dichotomy we observed in pathogen loading among a group of sites of the “East” immune class (see Materials and Methods section “Immune Classes” below). One site, MAN, is not included in the SFWMD survey, so we extracted values from the SFNRC data (which does sample at this location) for all dates when other sites were sampled.

### *Labyrinthula* spp. Load

#### qPCR Strain Specificity

Our group recently developed a new qPCR primer set in an effort to accurately quantify *Labyrinthula* spp. loading in seagrass tissue (Duffin et al., 2020). Notably, these primers were designed to target putatively pathogenic *Labyrinthula* spp. (*sensu*: Martin et al., 2016), irrespective of *Labyrinthula* phylotype or host seagrass species origin (e.g., the turtlegrass pathosystem hosts more than one phylotype). Our initial specificity test surveyed 37 *Labyrinthula* sp. isolates, reliably amplifying 100% of putatively pathogenic strains and only 15% of non-pathogenic strains (Duffin et al., 2020). In this current study, we were interested in providing more conclusive evidence that our protocol is indeed strongly biased toward pathogenic *Labyrinthula* spp. This was accomplished by evaluating several sets of additional criteria including: (1) relative Cq values of pathogenic vs. non-pathogenic isolates; (2) lower DNA concentrations (per reaction) reflective of field sample pathogen loading; (3) presence of “background” DNA; and, (4) simulation of a scenario where a non-pathogenic phylotype (specifically, one consistently amplifying in the previous study; Duffin et al., 2020) is found in a higher concentration compared to a pathogenic phylotype.

First, summary information was compiled for the average cycle quantification value (Cq, also referred to as Ct) of amplified strains across previous qPCR runs (conducted before and after publication of the Duffin et al., 2020 pilot study) with varying DNA template concentrations, to assess whether the qPCR assay amplified putatively pathogenic isolates more readily (i.e., at a lower Cq value, on average) than non-pathogenic isolates. In the pilot study, we tested strains using very high concentrations of DNA isolated from pure *Labyrinthula* sp. cultures. Thus, secondly, we adjusted the concentration of starting *Labyrinthula* sp. template DNA to 25 cells per reaction (converted from *Labyrinthula* sp. cells per mg dry seagrass tissue), to better match realistic concentrations in the field. This reflects a cell count greater than the load present in >95% (and within 1 SD of the highest load detected) of our FB-collected *T. testudinum* samples with detectable levels of the pathogen (this study). Third, we accounted for the possibility that non-specific binding may occur when using pure non-pathogenic *Labyrinthula* sp. cultures as the only template, so we introduced UltraPure™ Salmon Sperm DNA Solution (Invitrogen™) as background DNA in our diagnostic qPCR assays (i.e., to mimic host “background” DNA). Fourth, we evaluated bias for the case of pathogenic types being outnumbered by non-pathogenic. Specifically, we tested pathogenic isolate “8b” and non-pathogenic isolate “98b,” both of which reliably amplified in Duffin et al. (2020), but with notably different melt curve peaks at 76°C and 78.5°C, respectively, under several template DNA conditions: (1) pure *Labyrinthula* sp. culture at 1x concentration (2 μl 8b at 1x ≈ 646.8 cells per reaction; 2 μl 98b at 1x ≈ 1522.5 cells per reaction); (2) *Labyrinthula* sp. DNA equivalent of 25 cells per reaction with salmon sperm DNA to bring template volume to 20 ng total (i.e., 1 ng/μl reaction volume); and (3) both *Labyrinthula* sp. DNA loaded in a single reaction at a 1:70 ratio (~5.36 cells of 8b; ~375.2 cells of 98b per reaction), brought to 20 ng total with salmon sperm DNA. Each reaction was run in duplicate. 

#### Quantitative Real-Time PCR Procedure

##### DNA Extraction

Dry longitudinal half-leaf sections were pulverized to a fine powder with a tissue homogenizer. DNA was then extracted from a ~5 mg subsample using an Invisorb® Spin Tissue Mini Kit (Stratec Molecular, Berlin, Germany). The resulting eluent was purified using a OneStep™ PCR Inhibitor Removal Kit (Zymo Research, Irvine, CA, United States). Prior to assaying, DNA concentration was quantified *via* spectrophotometer, allowing sample standardization at 10 ng μl^−1^. See Duffin et al. (2020) for additional details.

##### Cell Counts/Standard Curve

We utilized the same standard curve protocol and template samples as in Duffin et al. (2020), derived from pure cultures of *Labyrinthula* sp. “E” isolate 8b, a strain isolated from and known to readily infect *T. testudinum* (Trevathan et al., 2011; Martin et al., 2016; Bishop et al., 2017). Briefly, cell concentrations from pure cultures were estimated using a Neubauer-improved hemocytometer, and the genomic DNA extracted as described above. This stock solution was then used to generate aliquots of DNA corresponding to *Labyrinthula* sp. cell concentrations (ranging from 320, 81, 5.0, 0.31, 0.02, and 0.0012 cells μl^−1^) for use in the standard curve for all qPCR runs.

##### qPCR Protocol

Primers (LabPathITS1-3F: 5'-CAA CTC AAT GAA TAT CTT GGT TTC C-3', and LabPathITS1-3R: 5’-CCG CTT ATT GAT ATG CTT AAA TTC-3') targeted the ITS region of the ribosomal RNA gene complex (Duffin et al., 2020). Quantitative PCR (qPCR) reactions were prepared with the following final concentrations: 1 ng μl^−1^ of DNA template, 0.025μM of each primer, 2.7 ng μl^−1^ of BSA, 1X of iTaq SYBR Green Supermix (Bio-Rad Laboratories, Hercules, CA, United States), and nuclease free water up to 20 μl. Reactions were run in triplicate on a CFX Connect thermal cycler (Bio-Rad) with the following cycle parameters: 5min at 95°C, followed by 45 rounds of 30 s at 95°C and 60 s at 63°C. Reactions were terminated with a melting curve analysis (65–95°C, at 0.5°C increments). Results are reported as the number of Labyrinthula spp. cells per mg starting seagrass tissue (dry weight, ~5 mg). See Duffin et al. (2020) for additional details.

#### Pathogen Load Trends in FB

##### Prevalence and Severity by Site

We defined pathogen prevalence as the proportion of individuals infected at a given site (presence/absence of *Labyrinthula* spp. cells) and pathogen severity as the number of cells present in a sample (averaged across three qPCR assay replicates) or group of samples (arithmetic mean across samples) in cells per mg dry weight of seagrass tissue.

##### Severity Classes

Pathogen severity values were averaged across the 10 individuals from each of the 15 sites and then grouped into three pathogen severity classes by first plotting a histogram of mean pathogen severity values by site, and then using visual cues to classify sites into three roughly equally sized groups. After verifying that the new severity classes were highly statistically different from one another, they were used in downstream analyses and visualizations.

### Host Immunity

#### Immunological Assay Procedures

Several common, well-studied immune activity enzymes were recently adapted for use with turtlegrass, including peroxidase (POX), exochitinase (EXOC), polyphenol oxidase (PPO), and a group of enzymes with lysozyme-like activity (LYS). The reader is referred to Duffin et al. (2020) for additional background and methodological details. Briefly, a small section (~0.1 g) of each *T. testudinum* blade (3rd rank, split longitudinally – with one half for use in qPCR, below) was homogenized and assayed. POX activity was measured using a modified guaiacol assay and presented as the change in absorbance at 470 nm min^−1^ μg^−1^ of total crude protein. EXOC activity was quantified using the enzymatic conversion of 4-methylumbelliferyl N-acetyl-β-D-glucosaminide into 4-methylumbelliferone (MU) and expressed as nmol of MU released per μg of total crude protein. PPO activity followed the conversion of L-DOPA to an oxidized dopachrome product and was quantified as a change in absorbance at 490 nm min^−1^ μg^−1^ of total crude protein. Finally, LYS activity was measured as the degree to which crude sample extract inhibited growth of *Micrococcus luteus*, given as percent inhibition per μg of total crude protein added as compared to uninhibited growth of the bacteria.

#### Immune Biomarker Levels by Site

Activity levels at each of the four biomarkers (POX, EXOC, PPO, and LYS) were compared among the 15 FB sites using a series of bubble plots. More specifically, bubbles were scaled following a linear relationship between mean sitewide biomarker level and bubble diameter, and then superimposed over a base map of the region to examine spatial patterns with respect to the geography of the bay.

#### Immune Classes

Multivariate analyses performed in PRIMER (v6) were utilized to group sites according to their overall immune status (simultaneously incorporating POX, EXOC, PPO, and LYS activity levels). First, we performed a hierarchical cluster analysis (CLUSTER routine), producing a dendrogram of similarity among sites based on immune status. This was followed by a similarity profile routine (SIMPROF), which is a permutation procedure intended to prevent over-dissection of subgroups and assess significance (Clarke et al., 2008). We also conducted non-metric multidimensional scaling (MDS) analysis with data from the four immune markers; the MDS groupings corroborated the site groupings of the CLUSTER analysis, so the results are not shown. These immune “classes” were used in downstream analyses and visualizations.

### Host Morphology and Immune Classes

We characterized turtlegrass morphology as a function of immune status by generating box plots to visually summarize mean leaf area (cm^2^) per shoot for each of three immune classes. While the original FHAP data set surveyed sites biannually (spring/fall), we detected no significant patterns between seasonal changes in leaf area and our factor of interest (immune grouping), so the results represent yearly averages of the two sampling points. The main box in the plot represents the interquartile range (IRQ) of the data, and the whiskers signify minimum and maximum spread of the data outside of the IRQ.

### Statistics

All statistical tests were performed with 95% confidence intervals (*α* = 0.05) on data that were tested for normality and computed using RStudio software (RStudio Team, 2018), unless otherwise stated. When plotting the immune activity data together for comparison, values were standardized through *Z*-score conversion in Microsoft Excel (v16.16.19). All error bars represent SEM, unless otherwise stated. Differences between groups were globally tested using a one-way ANOVA, followed by Tukey’s test for *post-hoc* analysis; alternatively, the non-parametric Kruskal-Wallis test was used when the data could not be transformed to meet assumptions of normality.

## Results

### qPCR Strain Specificity

A series of experiments were performed to further demonstrate that our qPCR assay (Duffin et al., 2020) preferentially amplifies pathogenic isolates of *Labyrinthula* spp. Pathogenic strain 8b and non-pathogenic strain 98b DNA, isolated from pure culture, both amplified in assays when high concentrations of either template were provided (Supplementary Figures S2A,B). However, average Cq values for 8b (20.16) were much lower than that of 98b (36.60), despite loading over twice as many cells in the 98b reaction as the 8b reaction. This pattern was representative of a larger trend: among qPCR assays with a standardized addition of *Labyrinthula* sp. DNA template (10 ng/μl input concentration), the average (±SEM) Cq value for pathogenic strains (26.54 ± 0.69, *n* = 18) was significantly lower (*p* < 0.001) than that of non-pathogenic strains (39.02 ± 1.12, *n* = 7).

Further, when templates were reduced to low concentrations (DNA isolated from ~25 *Labyrinthula* cells per reaction) and supplemented with background DNA (salmon sperm) to deter non-specific binding, the representative non-pathogenic strain failed to amplify in either replicate, while the pathogenic strain amplified readily (results not shown). Finally, both isolate DNA templates were analyzed simultaneously at a 1:70 ratio favoring the non-pathogenic strain and in the presence of background DNA. The amplified product featured a strong melt peak at 76°C, matching the expected peak temperature of 8b, with no indication of a secondary peak corresponding to 98b at 78.5°C (Supplementary Figure S2C).

### *Labyrinthula* spp. Load Trends in FB

#### Prevalence and Severity

**Figure 1:**
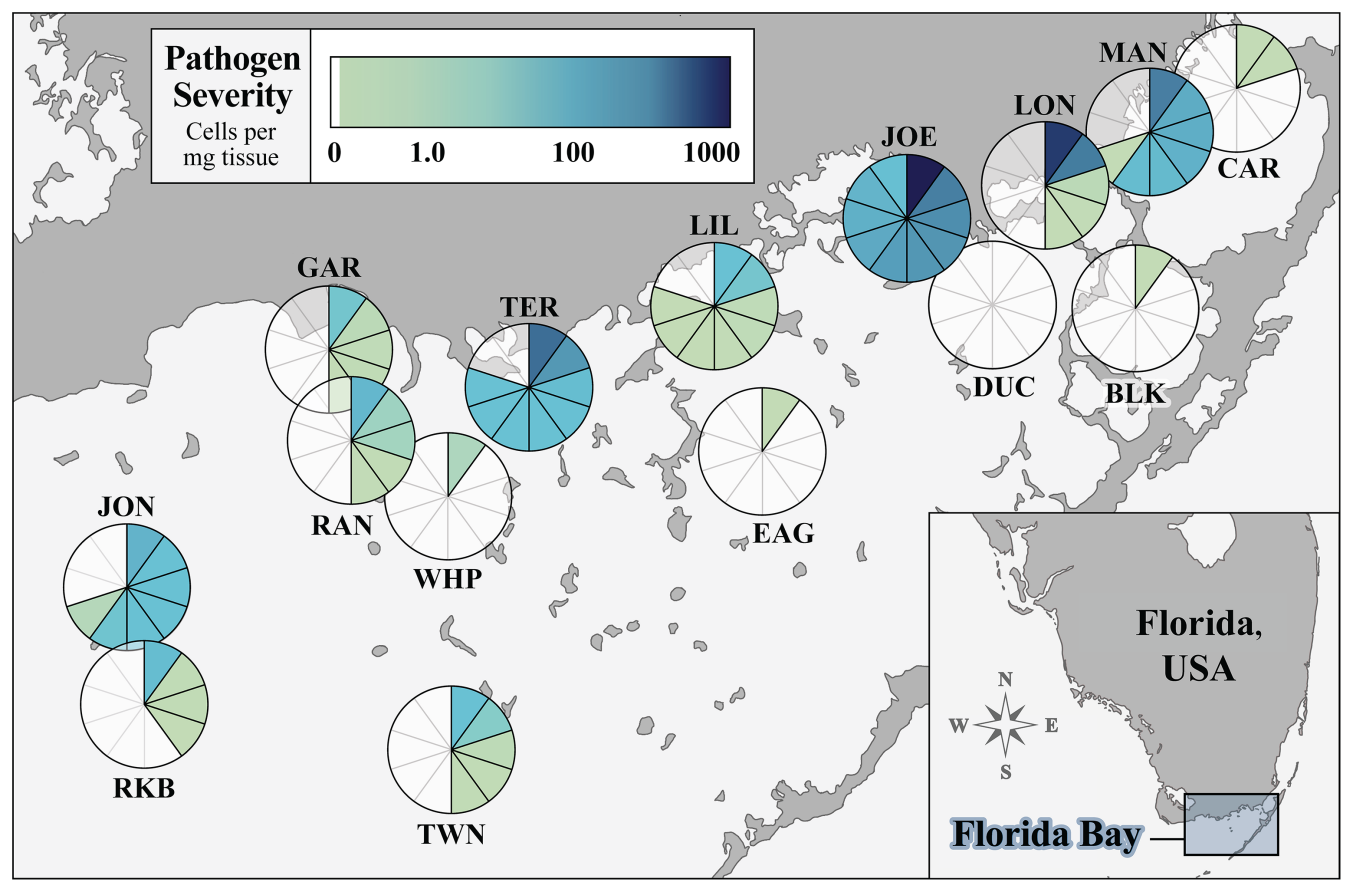
Prevalence and severity of *Labyrinthula* spp. in turtlegrass among 15 Florida Bay study sites. The pie chart layered over the location of a site represents disease prevalence (percent of samples out of 10 infected with quantifiable pathogen load) at that site. Each pie segment represents one *Thalassia testudinum* individual and is shaded according to pathogen severity (*Labyrinthula* spp. cells per mg dry weight of seagrass tissue). Sample size *n* = 10 individuals per site. Inset map shows study region relative to the mainland peninsula of Florida, United States.

We surveyed 15 sites in FB that captured the geomorphic, hydrological, and morphological variability of turtlegrass communities in the system. Figure 1 summarizes *Labyrinthula* spp. prevalence and severity values quantified in turtlegrass samples collected from each site, with each section of the pie representing one *T. testudinum* individual. Site prevalence, or percent of individuals with detectable levels of *Labyrinthula* spp. according to our qPCR assay, was 46% of individuals pooled across all 15 sites (69 of 150 samples). Site prevalence (*n* = 10 per site) ranged from 0% (DUC) to 100% (JOE; Figure 1; Supplementary Table S2). The severity of *Labyrinthula* spp. infection, or the quantified number of *Labyrinthula* spp. cells per mg dry seagrass tissue (i.e., putative pathogen load), was analyzed at both the individual level (pie wedge shading in Figure 1) and the site-wide level (Supplementary Table S2). The average load among all 150 samples collected was 40.05 cells mg^−1^; among those with detectable levels, the average was 87.07 cells mg^−1^. The maximum and minimum (non-zero) loading values among all individuals were 1009.08 and 4.89 × 10^−9^ cells mg^−1^, from JOE and CAR, respectively. Further, five of the top 10 most infected samples were collected from JOE; the remaining were from TER (*n* = 2), LON (*n* = 2), and BLK (*n* = 1). Site-wide severity averages ranged from 0.00 to 280.55 cells mg^−1^ (Supplementary Table S2). The pathogen was present with varying degrees of severity across all main geographic regions of the bay (western, central, and eastern; Figure 1).

#### Severity Classes

We further categorized sites into three classes according to their average (relative) pathogen severity for downstream analyses. Four sites (JOE, LON, TER, and MAN) fell under the “high” pathogen loading class, coded as “3”; seven sites (JON, RAN, RKB, LIL, TWN, GAR, and WHP) were characterized by “moderate” pathogen loading, coded as “2”; the remaining four sites (BLK, EAG, CAR, and DUC) had “low” (or zero) pathogen loading and were coded as the number “1” (Supplementary Table S2). The average (±SEM) pathogen loading of the severity classes were as follows: Individuals of Class 3 (high load; *n* = 40) harbored 143.14 ± 36.13 *Labyrinthula* spp. cells mg^−1^ tissue; plants in Class 2 (moderate load; *n* = 70) had an average of 4.03 ± 1.60 cells mg^−1^; and finally, individuals belonging to severity Class 1 (low load; *n* = 40) harbored 0.0017 ± 0.0013 cells mg^−1^. The overall differences between pathogen loading values across severity groups were highly significant (Kruskal-Wallis test; *p* < 0.001).

### Turtlegrass Immune Activity in FB

#### Biomarker Activity by Site

**Figure 2:**
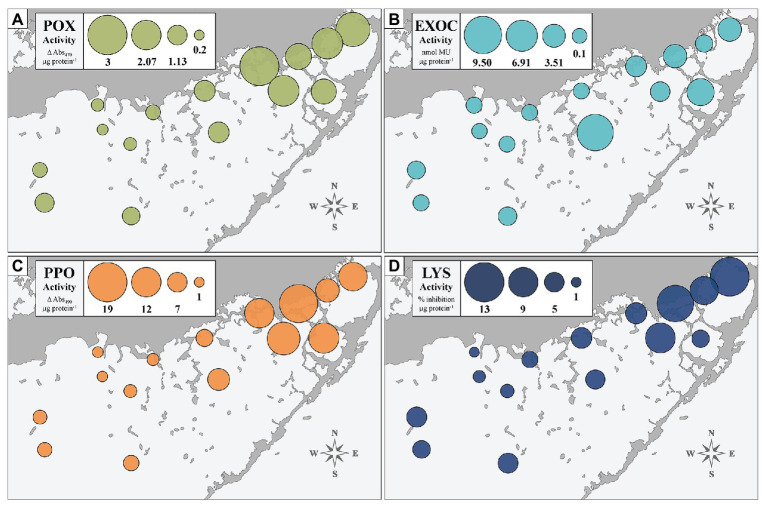
Immune enzyme activities of four biomarkers (POX, EXOC, PPO, and LYS: **A–D**) in turtlegrass among 15 Florida Bay study sites. Sizes of bubble plots layered over site locations represent magnitude of biomarker activity averaged across the 10 individuals collected at that site.

We assessed the immune status of FB-collected individuals at four biomarkers to provide a broad physiological perspective: POX, EXOC, PPO, and LYS. Figure 2 depicts activity of each of the four immune markers, averaged across individuals collected at each of the 15 sites (*n* = 10 per site). The size of the bubble represents relative activity at a given biomarker. Sites significantly differed from one another, overall, at the EXOC, POX, and PPO biomarkers at *α* = 0.01 (*p* < 0.0001), but the differences between sites were not significant at the LYS biomarker (*p* = 0.159). Generally, enzymatic trends followed an east-to-west gradient with low activity in the west and higher activity in the east (Figure 2). EXOC activity at the centrally-located site EAG represented an outlier to this trend, but was driven by only two individuals with notably high EXOC-substrate conversion rates (Figure 2B).

#### Immune Classes

We used hierarchical clustering to group sites together based on similarities at all four immune biomarkers (Supplementary Figure S3). The clustered divisions were supported by SIMPROF at *α* = 0.05; further, in an ANOVA analysis, the groupings differed significantly from one another at the EXOC, POX, and PPO biomarkers at *α* = 0.01 (*p* < 0.0001 each), and at the LYS biomarker at *α* = 0.05 (*p* = 0.042). The three distinct immune classes generated through these analyses (arranged lowest to highest response: A, B, and C) also distributed geographically, and thus further designated with the FB region they grouped in, where: A = “West” sites JON, TWN, LIL, and RKB; B = “Central” sites GAR, RAN, TER, and WHP; and C = “East” sites BLK, EAG, CAR, LON, JOE, DUC, and MAN (Supplementary Figure S3). Our multivariate clustering results reinforced trends seen at the site-level (Figure 2) in that the West and Central classes were more similar to one another than either were to the East. Importantly, this distinction was due to an elevation of biomarker activity in the East immune class as compared to both the West and Central classes (*p* < 0.05 at all pairwise comparisons except West-East LYS activity; Supplementary Figure S4).

### Morphology and Immunity

**Figure 3:**
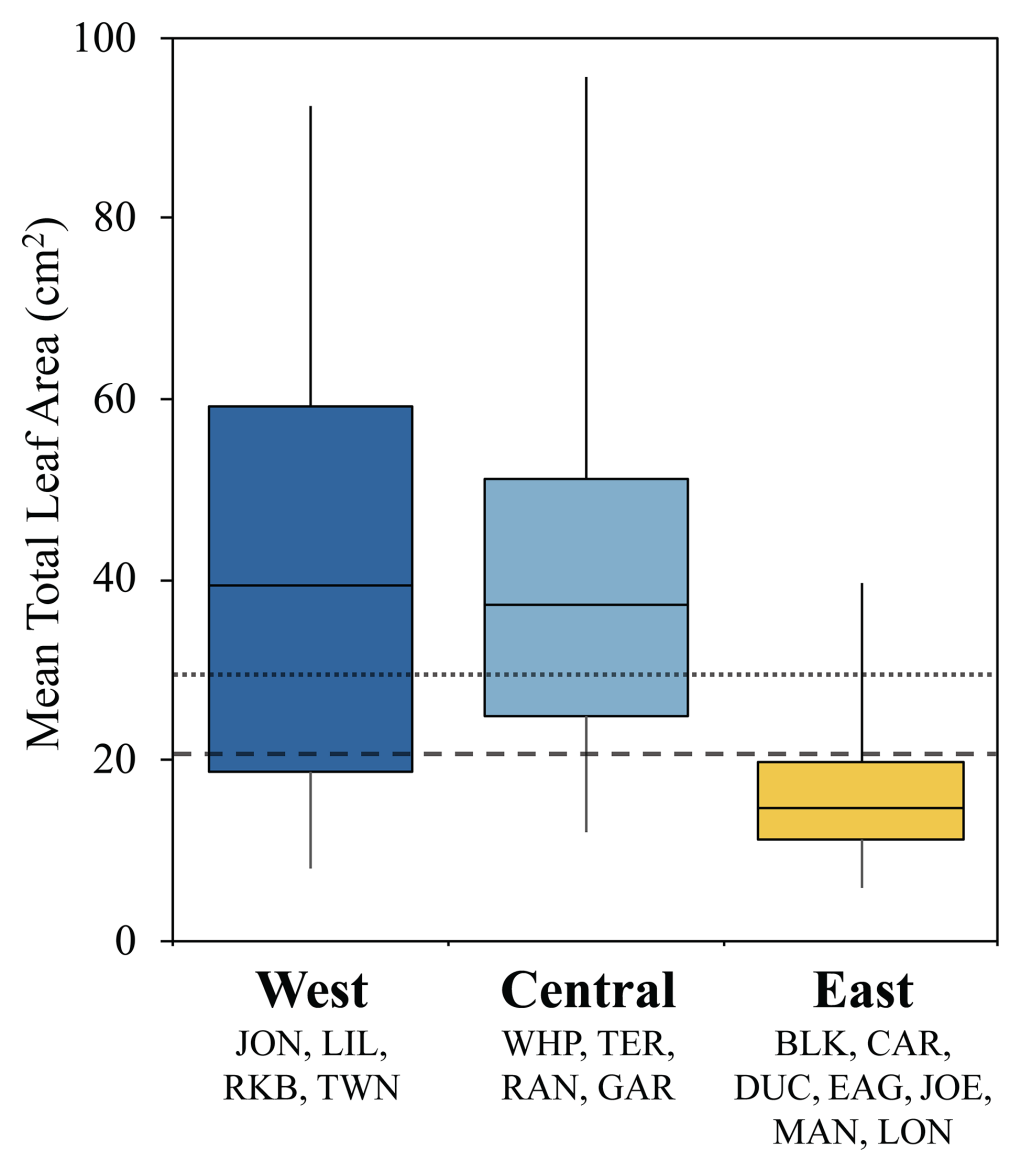
Box plots displaying turtlegrass mean leaf area per shoot (cm^2^) collected biannually from January 2010 to May 2015 as a function of immune class. Sample sizes as follows: West, *n* = 56; Central, *n* = 53; and East, *n* = 97. Boxplots represent the interquartile range (IRQ) centered around the median of the data. Whiskers signify the minimum and maximum spread of the data outside of the IRQ. Fine and coarse dashed lines represents the mean (29.26 cm^2^) and median (20.50 cm^2^) values across all sites, respectively. Data obtained through Fisheries Habitat Assessment Program (FHAP) survey database (M. Durako, pers. comm.).

Turtlegrass morphology varies remarkably across different basins of FB and is likely a plastic response to heterogeneous selective pressures across the bay (Hackney and Durako, 2004; Bricker et al., 2011). We examined potential relationships between *T. testudinum* morphology and immune activity by summarizing variability in mean leaf area (cm^2^) across sites clustered into the three immune classes (Figure 3). These differences were highly significant in a global ANOVA analysis (*p* < 0.0001); further, pairwise differences were significant between the East class and both the Central and West classes (Tukey’s *post-hoc*: *p* < 0.0001), but not between the West and Central classes (Tukey’s *post-hoc*: *p* = 0.841; Figure 3). Indeed, the interquartile range of the East class fell below both the mean (29.3 cm^2^) and median (20.5 cm^2^) values pooled across FB (fine and coarse dashed lines, respectively; Figure 3).

### Geography, Morphology, and Immunity

**Figure 4:**
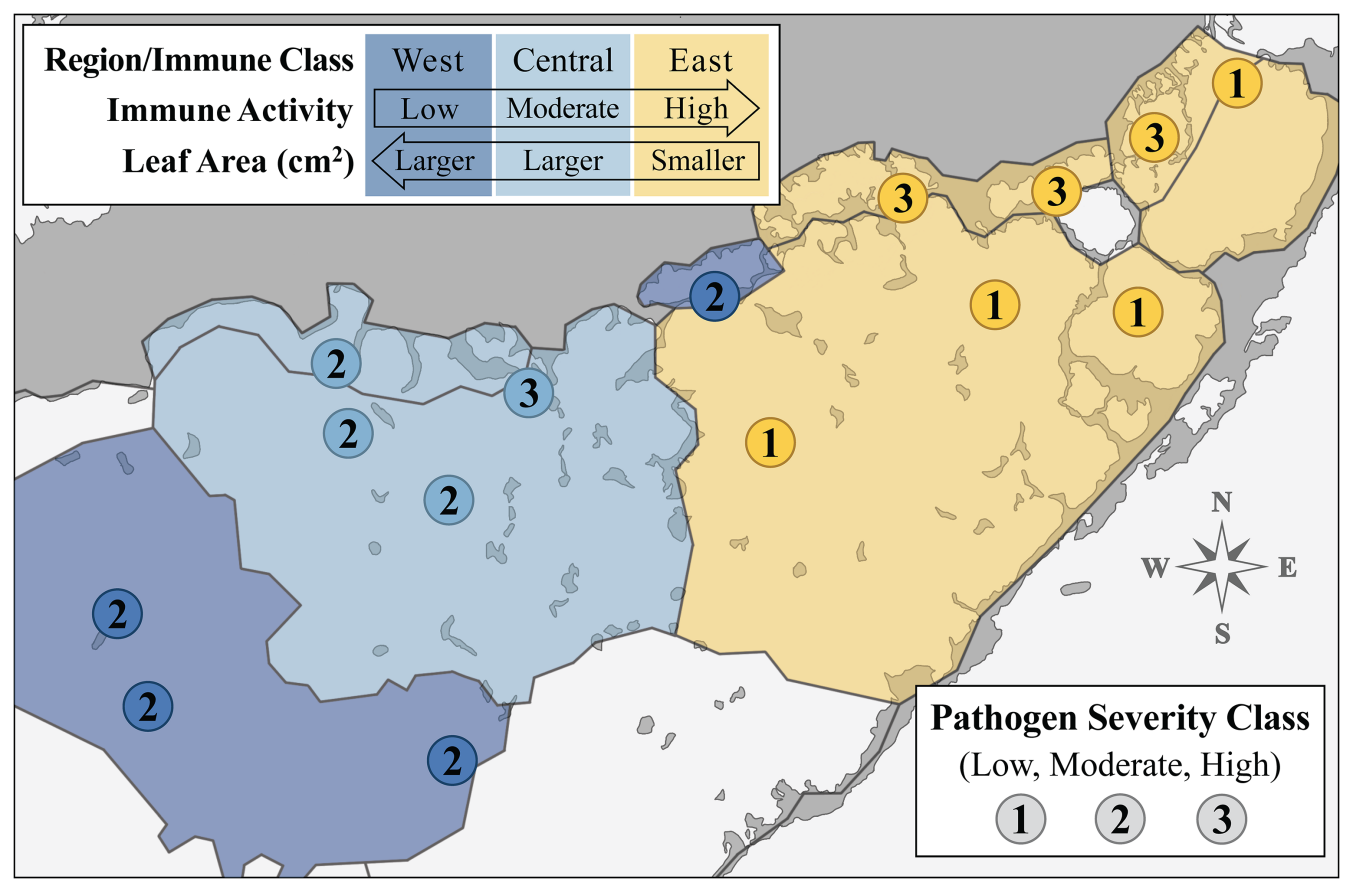
Map displaying site-level immune activity (color) and pathogen load (number label) classes layered over the biogeochemical segmentation of Florida Bay (RECOVER, 2010; Herbert et al., 2011) to effect designation of overall “immune response zones.” Numbers labeling each site represent pathogen severity class. Biogeochemical segments shaded according to associated immune class sites. Legend arrows indicate directional trends observed in composite enzyme measures (immune activity) and morphology (leaf area in cm^2^) across the spatial gradient of sampled Florida Bay regions.

Over the past several decades, several attempts have been made to partition the bay into discrete regions based on biogeochemical characteristics and ecological gradients (e.g., Wanless and Tagett, 1989; Boyer et al., 1999; Bricker et al., 2011; Briceño et al., 2013). We reviewed and compared these biogeographical/ecological models to the spatial patterns observed in our data (Figures 1, 2). One model, a projected reorganization of seagrass communities based on changes in freshwater inflow described in Herbert et al. (2011), largely coincided with the geographical distribution of the immune classes we established in this study (Supplementary Figure S3). Superimposing our data over Herbert et al. (2011), we created a layered map of FB (Figure 4) where biogeochemical zone delineations were outlined in black, and shaded according to the immune classes that coincided with these regions (West, Central, and East; shaded in dark blue, light blue, and yellow, respectfully; Figure 4), now representing “immune zones.” A third layer of data, pathogen severity class, was incorporated into the zone map by adding the numerical label for each pathogen severity class (low, moderate, and high severity: “1,” “2,” and “3,” respectively) at each site location (Figure 4).

Generally, immune activity increased from west to east in FB, in a roughly inverse relationship with leaf area (cm^2^), which decreased along the same gradient (Figure 4). Further, eight of the nine sites within the West or Central zone had moderate (“2”) pathogen loading, excluding site TER in the Central zone (Figure 4). This is in contrast with pathogen load in the East zone, where sites always fell within one of the two extremes; the three sites closest to the mainland (JOE, LON, and MAN) all had high (“3”) loading, whereas the four sites more distal to the shoreline (EAG, DUC, BLK, and CAR) had low (“1”) loading (Figure 4).

### Antecedent Salinity and Pathogen Load

**Figure 5:**
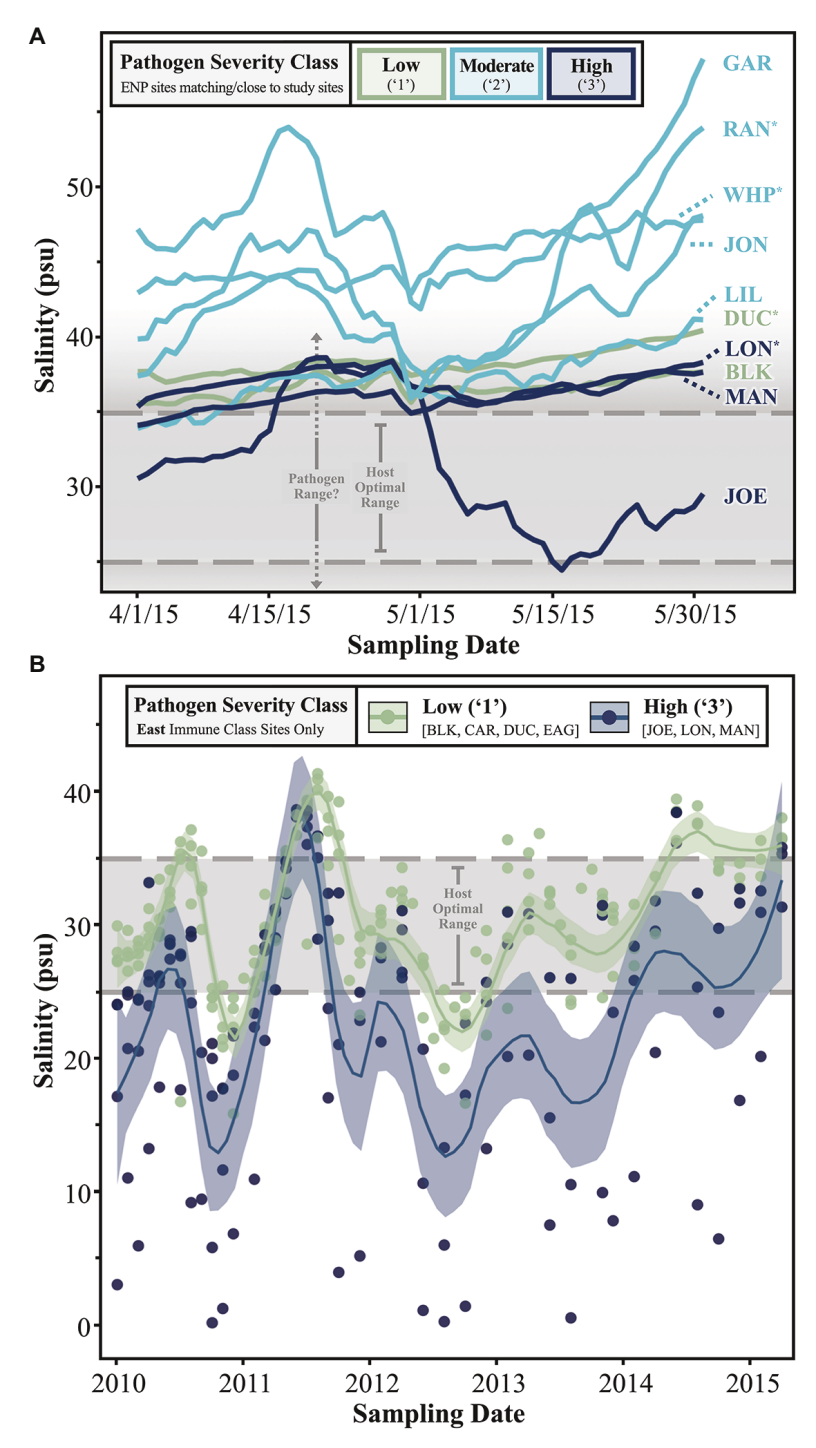
Line graphs depicting trends in **(A)** short- and **(B)** long-term salinity measurements collected from either **(A)** all ENP sites in close proximity to our study sites or **(B)** permanent FHAP transects/our study sites falling within the “East” immune class. In part **(A)**, the data represents ENP station sampling points but are named according to our study sites/FHAP basins they pair with. Asterisks (*) denote study sites with nearby, but not perfectly complementary, ENP stations. See Supplementary Figure 1 for details.

One driving aim of CERP is to manipulate freshwater output from the South Florida watershed to restore historic conditions which favored a more diverse species assemblage in seagrass communities (Everglades Forever Act, 1994; Florida Forever Act, 2000; Water Resources Development Act, 2000). Thus, we were interested in relating temporal patterns of salinity to *Labyrinthula* spp. loading in host turtlegrass tissue. First, we examined daily salinity trends over a 2-month period leading up to the 2015 sampling date (Figure 5A). Sites with moderately severe (“2”) pathogen load generally experienced the most saline conditions in the 2 months leading up to the sampling period, while sites falling in the low and high pathogen severity classes (“1” and “3,” respectively) experienced lower salinities (Figure 5A). The majority of sites spent most of April and May of 2015 outside the host optimal salinity range (25–35 psu), and likely the pathogen salinity range as well (see Discussion section). One site with high pathogen load, JOE, stands out due to the significant dip in salinity beginning around May 1, 2015, approximately 1 month before tissue collection (Figure 5A). This site is also distinct in terms of pathogen load, even among other sites of the “High” pathogen severity class; pathogen prevalence at JOE was 100% and average pathogen severity was nearly double that of the next highest site, LON (280.6 ± 88.8 vs. 122.8 ± 86.7 cells mg^−1^, respectively; Supplementary Table S2).

Finally, we further investigated the stark dichotomy displayed by the pathogen severity classes of sites in the East immune zone, noting that “High” pathogen severity-grouped sites in this zone were located within semi-enclosed coastal basins, while “Low” sites were all more distal to the coast and in relatively open water (Figure 4). Specifically, we examined longer-term salinity trends in the East zone by plotting monthly salinity data by site from 2010 to 2015 (color coded by pathogen severity class), with local regression (loess) smoothing lines defining average trends shared by sites in either pathogen severity class (Figure 5B). While salinity fluctuations in “Low” severity class sites were often confined within the upper and lower margins of the host optimal range, conditions in “High” severity class sites often dipped well below the lower threshold optima of 25 psu (Figure 5B). Extreme hyposaline conditions (between 0 and 10 psu) were not uncommon among high-load coastal sites JOE, LON, and MAN, but never occurred during the ~4.5 year span among offshore low-load sites BLK, CAR, DUC, and EAG (Figure 5B). In addition to experiencing frequent extreme hyposaline conditions, sites of the “High” severity class experienced greater fluctuations in salinity, with hypersaline conditions comparable to that of the “Low” severity class sites (Figure 5B). For example, we note extreme fluctuation amplitudes (trough to crest) in high-load sites measuring between ~20 and ~38 over an approximate 6 month period (late 2010 to mid 2011; Figure 5B).

## Discussion

Florida Bay turtlegrass meadows are characterized by a well-documented phenotypic gradient with larger-bodied high-density *T. testudinum* shoots in the West and Central regions, and smaller low-density shoots in the East (this study; Bricker et al., 2011). We leveraged this, along with the known environmental gradients and homogeneous distribution of genotypes across the region, for comparative purposes. Our composite measure of host immune metrics displayed a comparable but opposing gradient to that of phenotype, exhibiting the lowest enzymatic responses in the West and highest in the East. We further characterized FB turtlegrass by surveying both pathogenic *Labyrinthula* spp. prevalence and load *in situ*; to our knowledge, this component of the study was the first of its kind for *T. testudinum*. Additionally, we build off of a previous study (Duffin et al., 2020) in providing more evidence that the primers used also serve as a universal qPCR-based method to quantify pathogenic *Labyrinthula* spp. loading in other seagrass species. Specifically, we confirmed that qPCR bias heavily favors seagrass-pathogenic clade phylotypes (*sensu*: Martin et al., 2016) when used in an ecological context (i.e., when at realistic concentrations, and with ample background DNA – as from host tissue; Supplementary Figure S2), even when outnumbered (1:70) by a non-pathogenic phylotype. Overall, *Labyrinthula* spp. were detected at 14 of the 15 sites, showed a prevalence of 46% for individual shoots, and exhibited a strong spatial pattern related to host immunity and morphology, and the ecophysiography of FB. Below, we unite our data observations with previous FB characterizations of resource availability and resistance-tolerance theory, deconstructing this complex pathosystem into a simple, conceptual model that serves as a framework for future hypothesis-driven work. In summary, we posit that FB turtlegrass defenses present with a strategy of constitutive immune resistance in more stressful environments but adopt a strategy of tolerance when resources are more plentiful.

### Immunity – Geography and Morphology

Immune enzyme activities were used to reflect the functional status of host defense pathways. Our results showed spatially coherent clusters that were based upon enzyme (POX, EXOC, PPO, and LYS) activities: lowest in western and central FB and highest in the east (Figure 2 and Supplementary Figure S4; hereafter: West, Central, and East classes). Because immune status aligned well with the ecophysiographic delineations described in Herbert et al. (2011), we interpreted them as “immune response zones” (Figure 4) with a linkage between plant immunity and the biogeochemistry of the bay. It was noted that individuals of the East immune class had much lower total leaf area than individuals of either Central or West regions (Figure 3), as expected from the east-west gradient described by Bricker et al. (2011). The driver of modest differences between the West and Central zones remains unclear but may be a consequence of gradient sampling.

### Defense Strategies – Resistance and Tolerance

Tolerance and resistance are two basic strategies plants have when facing disease: the term “tolerance” describes a host’s ability to minimize the impact of infection on fitness without specifically reducing pathogen load in host tissue, while “resistance” characterizes the plant’s ability to mount a response that actively limits pathogen reproduction (Råberg, 2014; Pagán and Garcia-Arenal, 2018). There can be trade-offs between the two strategies and they are not necessarily mutually exclusive (Pagán and Garcia-Arenal, 2018). That said, the existence of a disease tolerance strategy appears not to have been evaluated critically for any seagrass – including this study. Our data do not allow us to quantify or conclude a strategy of tolerance, but we use the concept to relate spatial patterns in our survey and to generate testable hypotheses. The composite immune metric (assimilation of all four enzyme activities) used in this study suggests that smaller (more eastern) individuals may be allocating more resources to constitutive resistance, while larger (more western) individuals appear to tolerate mostly moderate levels of infection.

Importantly, resistance and tolerance strategies yield different selective pressures (as only the former directly reduces pathogen reproduction, or infectivity), while theory predicts the two may coevolve with one another, and with pathogen virulence, in complex ways (Best et al., 2009; Carval and Ferriere, 2010; Little et al., 2010). Since tolerance can increase prevalence, it may have special relevance to disease spread in times of climate change (Kutzer and Armitage, 2016). Both strategies can be evidenced from classical reaction norm studies, relative to uninfected status (i.e., underlying vigor), where fitness is a function of pathogen loading or disease severity (Stowe et al., 2000; Baucom and de Roode, 2011). While we did not include such a study, two of our markers were consistent with an inducible response to *Labyrinthula* sp. infection, and all four markers were often associated with pathogen defenses (Duffin et al., 2020). We, therefore, interpret our immune composite measures as broadly reflecting underlying resistance. Our claim of tolerance remains conjectural, however, and we provide no alternative indirect measure aside from size differences (e.g., tolerance is often achieved through compensatory growth; Rausher, 2001; Baucom and de Roode, 2011) and consistent pathogen loading.

**Figure 6:**
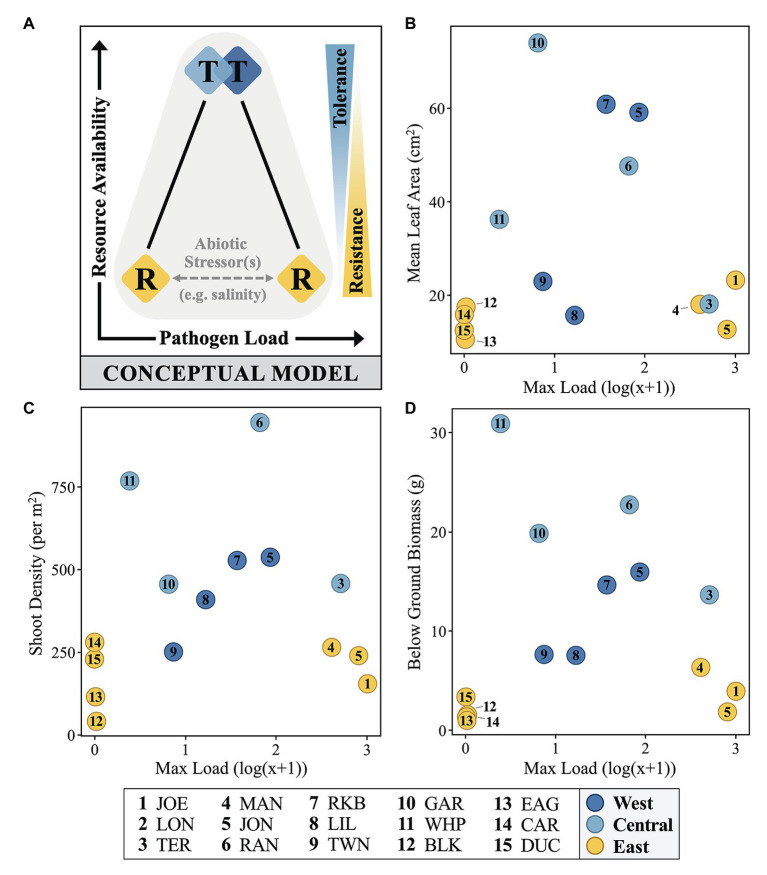
A conceptual model **(A)** and selected fitness metrics **(B–D)** relating immunostatus, *Labyrinthula* pathogen loading and host phenotype of FB turtlegrass. In part **(A)**, colored diamonds represent field measures of mean immune zone responses adopting a strategy somewhere along the tolerance (“T”) and resistance (“R”) continuums (represented as blue/yellow wedge gradients, right). Measures of pathogen load and resource availability increase along the *x*- and *y*-axes, respectively. Influence of abiotic stressors (represented by gray horizontal dashed line), such as salinity stress, is suggested to play a role in determining pathogen load in hosts presenting with a strategy of resistance (“R”). In parts **(B–D)**, data are plotted along similar *x*- and *y*-axes as in the conceptual model; here, pathogen load is represented as the log(x + 1) value of the most infected individual at a given site and resource availability is substituted with three measures of morphological data that serve as reasonable proxies for plant fitness: mean leaf area (cm^2^), shoot density (per m^2^), and below ground biomass (g).

To explain the temporospatial relationship between immunostatus, pathogen loading, and host phenotype across the FB seascape, we introduce a simple, testable conceptual model treating resistance and tolerance as opposite (though not mutually exclusive) ends of a resource availability gradient (*y*-axis; Figure 6A), with the former subject to environmental perturbation. Field measures of mean immune zone responses (diamonds, colored as in Figure 4) are shown as a function of resources and load, but also in relation to posited defense strategies as they reflect relative directional relationships (right *y*-axis; Figure 6A) to resource availability. The dichotomy of load levels in the East zone (yellow) sites as either very low or very high suggests the presence of a significant but unidentified factor(s) contributing to this pattern (see below). Though other studies have shown differing relations to environmental resources (Kutzer and Armitage, 2016), recent empirical work by Zeller and Koella (2017) indicates that challenging environments can actually lead to higher levels of parasite defense, but also that higher resources can lead to the evolution of tolerance. For plants, especially longer-lived types, the expectation of high resource environments generally favoring tolerance is not new (Maschinski and Whitham, 1989), but also not universal. Although we chose leaf area as a measure of size to confirm the earlier work describing the size gradient across FB (Bricker et al., 2011), a *post-hoc* look at several fitness metrics yielded the same basic pattern, consistent with our conceptual model (Figures 6B–D). This relation was especially notable for below-ground biomass (Figure 6D), reinforcing the notion that resource availability is a key factor.

From an antagonistic coevolutionary perspective (i.e., an evolutionary arms race; Roy and Kirchner, 2000; Rausher, 2001; Kutzer and Armitage, 2016), the differential success of resistance between the inshore and offshore sites in the East zone (Figure 4) could reflect conditions conducive for the evolution of a more virulent strain at inshore sites. Regardless, it is clear that future work on tolerance and *Labyrinthula* sp. evolution has the potential to predict where and when more virulent strains might emerge, provide a mechanistic explanation for outbreaks, or inform conservation efforts that might target tolerance over resistance (e.g., Rohr et al., 2010; but see Carval and Ferriere, 2010).

Although not designed to evaluate tolerance *per se*, studies by Brakel et al. (2014, 2019) invoke tolerance to SWD by suggesting that intra-leaf and/or younger-leaf growth compensates for lesion size, though to what extent this is adaptive is unclear. For example, in Brakel et al. (2014), it appears any benefit to increased growth could be offset by the decreased rate of emerging leaves relative to non-infected shoots. In Brakel et al. (2019), their measure of net leaf growth outpacing lesion size appears to show compensatory growth within individuals, but a measure of growth for uninfected individuals (i.e., vigor) is not provided, and possible loss of leaf function above the lesion (Steele et al., 2005) is not addressed. The effect also appears to diminish with infection intensity. Finally, Brakel et al. (2017) found that growth did not keep pace with lesion extent. In summary, it seems that eelgrass may be capable of partial compensation, but it is unclear under what conditions, or what effect this might have on below-ground reserves and thus long-term fitness.

### FB Salinity Trends

Florida Bay is part of the world’s largest restoration initiative with projected costs of over a billion dollars; one of the main tools under management control is freshwater flow into FB [U.S. Army Corps of Engineers and U.S. Department of the Interior (USACE-DOI), 2015]. While earlier studies do not identify specific phylotypes of *Labyrinthula* sp., it was recognized early on that eelgrass wasting disease of the 1930s was less common in lower salinity meadows (Muehlstein, 1989; Burdick et al., 1993). Similarly, both cultured isolates and controlled mesocosm observations show better growth or disease progression above about ~10–25 psu, but below ~40–50 psu for both eelgrass (Young, 1943; Pokorny, 1967; Short et al., 1987; Muehlstein et al., 1988; McKone and Tanner, 2009) and turtlegrass pathosystems (Martin et al., 2009; Trevathan et al., 2011; Bishop et al., 2017). However, to our knowledge, more detailed studies of salinity effects within the turtlegrass pathosystem are lacking.

Florida Bay is a seasonally hypersaline, reverse estuary with spatiotemporally variable salinities driven largely by direct rainfall, runoff, shallow bathymetry, and extreme climatic events (i.e., hurricanes and droughts; Kelble et al., 2007; Cole et al., 2018). Annual trends in FB salinity indicate that there are predictable, widespread oscillations between hypersaline conditions in early summer and, in the coastal basins, hyposaline conditions in the early-winter (Kelble et al., 2007), so it is not surprising that daily measurements antecedent to our early June 2015 sampling were between 35 and 55 psu, above the optimal range of turtlegrass (Figure 5A). One site, JOE, experienced a rapid salinity decline within 30 days of our collection, likely the result of an acute runoff event from the Everglades (Kelble et al., 2007; Sullivan et al., 2014). Interestingly, the sampling site in Joe Bay also experienced the highest pathogen loads, in both prevalence and severity of *Labyrinthula* spp. infection. Superficially, it might be surprising that the only site where salinity fell within the host optimal range during the time of collection also experienced the highest infection. However, high salinity is equally or more likely to inhibit pathogen growth and/or transmission (e.g., Martin et al., 2009; Bishop et al., 2017), and to the extent that pathogens can respond more quickly than host resistance efforts, short-term salinity could explain the small-scale patterns we documented.

Salinity stress might also play a role in the relative success of resistance vs. tolerance strategies adopted by different turtlegrass populations of FB over longer temporal scales. The 5-year view of monthly salinity trends among sites of the East immune response zone revealed that locations with highest pathogen loads also experienced dramatic fluctuations between moderate saline conditions (i.e., within turtlegrass and *Labyrinthula* sp. optimal range) and periods of extreme hyposalinity (Figure 5B). Thus, chronic environmental stress may impair the immune response of the host. Specifically, we note that the relatively enhanced immune response of East zone individuals appears to have been successful at sites where longer-term hyposalinity stress events were minimal (more off-shore sites), but small-bodied high-immune activity individuals that routinely experienced hyposaline stress (i.e., more coastal basins) succumbed to *Labyrinthula* spp. infection (Figure 5B). From this perspective, the results generally suggest that plant morphology, immune status, and hyposalinity events interact to drive differences in the host susceptibility to pathogen loading across FB. Finally, despite our focus on salinity (due to its historic influences in the study system and compelling fit with our data), we further emphasize that this stressor represents just one of many potential abiotic drivers modulating defense strategies and wasting disease in FB turtlegrass.

### Conclusion

It has been nearly a century since the massive 1930’s north Atlantic eelgrass wasting events attributed to *Labyrinthula* sp., yet, we struggle to predict or describe such outbreaks (Sullivan et al., 2013, 2018). This can be attributed in part to the opportunistic nature of some *Labyrinthula* spp. – a term that may reflect more about our lack of knowledge regarding the specifics and/or variety of mechanisms affecting hosts and pathogens (e.g., emergent properties: Bull et al., 2012; Egan and Gardiner, 2016), with the latter serving only as an operational definition (Pirofski and Casadevall, 2012). Nonetheless, it is important to move forward by uncovering factors affecting disease dynamics in foundational species such as turtlegrass. Toward this end, our new tools for qPCR and resistance metrics show promise for seagrass-pathosystem studies. In addition to the strong morphological plasticity documented across the complex environmental gradient of FB, turtlegrass itself appears to possess significant physiological plasticity in immunostatus and pathogen defense strategies. Our data also hint at the role of environmental quality on both host defenses and shorter-term factors that appear to drive pathogen transmission/infection dynamics capable of overrunning host defenses. Importantly, our results were inconsistent with classic density-dependent explanations for disease prevalence, indicating that these host-pathogen dynamics are far more complex than just conditions that affect transmission. While resistance is often the metric of choice in defense studies, we argue that investigations of tolerance could provide needed perspective for disease dynamics in terms of pathogen prevalence and evolution (e.g., virulence) in a changing climate.

## Data Availability Statement

The raw data supporting the conclusions of this article will be made available by the authors, without undue reservation.

## Author Contributions

CR initiated and acquired funding for this project. PD, DM, and CR conceptualized the ideas that drove the manuscript. PD and DM led methodological advancements in the qPCR assay. Pure *Labyrinthula* culture collections were carried out by DM. PD acquired samples, curated sample data, and led the formal analysis and investigation, with help from DM, BF, and CR. Environmental data acquisition and multivariate analyses relied on the expertise of BF. Visualization of data was primarily achieved by PD and BF, with input from DM and CR. All authors contributed to the article and approved the submitted version.

### Conflict of Interest

The authors declare that the research was conducted in the absence of any commercial or financial relationships that could be construed as a potential conflict of interest.

## Footnotes

^1^ https://www.sfwmd.gov/science-data/dbhydro
