# Corrigendum: Spatial Patterns of *Thalassia testudinum* Immune Status and *Labyrinthula* spp. Load Implicate Environmental Quality and History as Modulators of Defense Strategies and Wasting Disease in Florida Bay, United States

**DOI:** 10.3389/fpls.2022.877673

**Published:** 2022-03-21

**Authors:** Paige Duffin, Daniel L. Martin, Bradley T. Furman, Cliff Ross

**Affiliations:** ^1^Department of Biology, University of North Florida, Jacksonville, FL, United States; ^2^Department of Genetics, University of Georgia, Athens, GA, United States; ^3^Florida Fish and Wildlife Research Institute, Florida Fish and Wildlife Conservation Commission, St. Petersburg, FL, United States

**Keywords:** immunocompetence, host-pathogen interactions, hyposalinity stress, opportunistic pathogens, environmental fluctuation, anthropogenic influences, resistance, tolerance

In the original article, there was an error. The primer set sequences we originally provided did not accurately reflect the primer set sequences we used to conduct the study. The original study referenced, however, does contain the correct sequence (Duffin et al., 2020).

A correction has been made to *Materials and Methods* > Labyrinthula *spp. Load* > *Quantitative Real-Time PCR Procedure* > *qPCR Protocol*, paragraph one:

Primers (LabPathITS1-3F: 5′-CAA CTC AAT GAA TAT CTT GGT TTC C-3′, and LabPathITS1-3R: 5′-CCG CTT ATT GAT ATG CTT AAA TTC-3′) targeted the ITS region of the ribosomal RNA gene complex (Duffin et al., 2020). Quantitative PCR (qPCR) reactions were prepared with the following final concentrations: 1 ng μl^−1^ of DNA template, 0.025 μM of each primer, 2.7 ng μl^−1^ of BSA, 1X of iTaq SYBR Green Supermix (Bio-Rad Laboratories, Hercules, CA, USA), and nuclease free water up to 20 μl. Reactions were run in triplicate on a CFX Connect thermal cycler (Bio-Rad) with the following cycle parameters: 5 min at 95°C, followed by 45 rounds of 30 s at 95°C and 60 s at 63°C. Reactions were terminated with a melting curve analysis (65–95°C, at 0.5°C increments). Results are reported as the number of *Labyrinthula* spp. cells per mg starting seagrass tissue (dry weight, ~5 mg). See Duffin et al. (2020) for additional details.

In the original article, there were a few errors in reporting concentrations of *Labyrinthula* strains used in the qPCR specificity assays. Specifically, there were three locations within a single sentence where “ml” was inadvertently used instead of “μl.”

A correction has been made to *Materials and Methods* > Labyrinthula *spp. Load* > *qPCR Strain Specificity*, paragraph two:

First, summary information was compiled for the average cycle quantification value (Cq, also referred to as Ct) of amplified strains across previous qPCR runs (conducted before and after publication of the Duffin et al., 2020 pilot study) with varying DNA template concentrations, to assess whether the qPCR assay amplified putatively pathogenic isolates more readily (i.e., at a lower Cq value, on average) than non-pathogenic isolates. In the pilot study, we tested strains using very high concentrations of DNA isolated from pure *Labyrinthula* sp. cultures. Thus, secondly, we adjusted the concentration of starting *Labyrinthula* sp. template DNA to 25 cells per reaction (converted from *Labyrinthula* sp. cells per mg dry seagrass tissue), to better match realistic concentrations in the field. This reflects a cell count greater than the load present in >95% (and within 1 SD of the highest load detected) of our FB-collected *T. testudinum* samples with detectable levels of the pathogen (this study). Third, we accounted for the possibility that non-specific binding may occur when using pure non-pathogenic *Labyrinthula* sp. cultures as the only template, so we introduced UltraPure™ Salmon Sperm DNA Solution (Invitrogen™) as background DNA in our diagnostic qPCR assays (i.e., to mimic host “background” DNA). Fourth, we evaluated bias for the case of pathogenic types being outnumbered by non-pathogenic. Specifically, we tested pathogenic isolate “8b” and non-pathogenic isolate “98b,” both of which reliably amplified in Duffin et al. (2020), but with notably different melt curve peaks at 76°C and 78.5°C, respectively, under several template DNA conditions: (1) pure *Labyrinthula* sp. culture at 1x concentration (2 μl 8b at 1x ≈ 646.8 cells per reaction; 2 μl 98b at 1x ≈ 1522.5 cells per reaction); (2) *Labyrinthula* sp. DNA equivalent of 25 cells per reaction with salmon sperm DNA to bring template volume to 20 ng total (i.e., 1 ng/μl reaction volume); and (3) both *Labyrinthula* sp. DNA loaded in a single reaction at a 1:70 ratio (~5.36 cells of 8b; ~375.2 cells of 98b per reaction), brought to 20 ng total with salmon sperm DNA. Each reaction was run in duplicate.

The authors apologize for this error and state that this does not change the scientific conclusions of the article in any way. The original article has been updated.

## Publisher's Note

All claims expressed in this article are solely those of the authors and do not necessarily represent those of their affiliated organizations, or those of the publisher, the editors and the reviewers. Any product that may be evaluated in this article, or claim that may be made by its manufacturer, is not guaranteed or endorsed by the publisher.
