# Supplementary material for: Spatial Patterns of Thalassia testudinum Immune Status and Labyrinthula spp. Load Implicate Environmental Quality and History as Modulators of Defense Strategies and Wasting Disease in Florida Bay, United States
Source: Front Plant Sci. 2021 Feb 5;12:612947. doi: 10.3389/fpls.2021.612947 (PMC7892610; doi:10.3389/fpls.2021.612947)
Supplement: Supplementary file 1 [file Data_Sheet_1.pdf]

Supplementary Material

1 Supplementary Figures and Tables

1.1 Supplementary Tables

Supplementary Table 1. Site information (full site name, coded abbreviation, collection date, and decimal coordinates) for the 15 locations surveyed in Florida Bay during May of 2015.

Study Site/ FHAP Basin	Site Code	Collection (mm/dd/yy)	Coordinates	
			Latitude	Longitude
Johnson Key Basin	JON	05/26/15	25.04247	-80.91482
Rabbit Key Basin	RKB	05/30/15	25.00242	-80.90010
Garfield Bight	GAR	05/25/15	25.15048	-80.80922
Rankin Lake	RAN	05/25/15	25.12138	-80.80288
Whipray Basin	WHP	05/26/15	25.09142	-80.75478
Twin Key Basin	TWN	05/30/15	24.97767	-80.75352
Terrapin Bay	TER	05/26/15	25.14037	-80.71612
Little Madeira Bay	LIL	05/27/15	25.17517	-80.62692
Eagle Key Basin	EAG	05/27/15	25.11797	-80.59972
Joe Bay	JOE	05/31/15	25.22447	-80.53658
Duck Key	DUC	05/29/15	25.17707	-80.49157
Long Sound	LON	05/28/15	25.22737	-80.46167
Blackwater Sound	BLK	05/28/15	25.17405	-80.42308
Manatee Bay	MAN	05/29/15	25.25103	-80.41517
Card Sound Bridge	CAR	05/29/15	25.27355	-80.37458

Supplementary Table 2. Pathogen prevalence (percent of samples infected with quantifiable *Labyrinthula* spp. load) and severity (average and standard error of *Labyrinthula* spp. cells per mg dry weight of seagrass tissue from individuals collected) for each collection site within Florida Bay. Cell shading reflects proportional magnitude of the parameter at that site. Sample size n = 10 individuals per site.

**Bay Site**	**Pathogen Prevalence** (% infected)	**Pathogen Severity** (cells per mg tissue)		**Severity Class**
		Mean	SE	
JOE	100	280.6	88.8	3
LON	50	122.8	86.7	3
TER	80	86.27	52.9	3
MAN	70	82.98	39.2	3
JON	70	13.39	8.1	2
RAN	50	7.000	6.4	2
RKB	40	3.641	3.6	2
LIL	80	2.197	1.6	2
TWN	50	1.155	0.7	2
GAR	50	0.667	0.5	2
WHP	10	0.143	0.1	2
BLK	10	0.005	0.0	1
EAG	10	0.002	0.0	1
CAR	20	0.000	0.0	1
DUC	0	0.000	0.0	1

1.2 Supplementary Figures

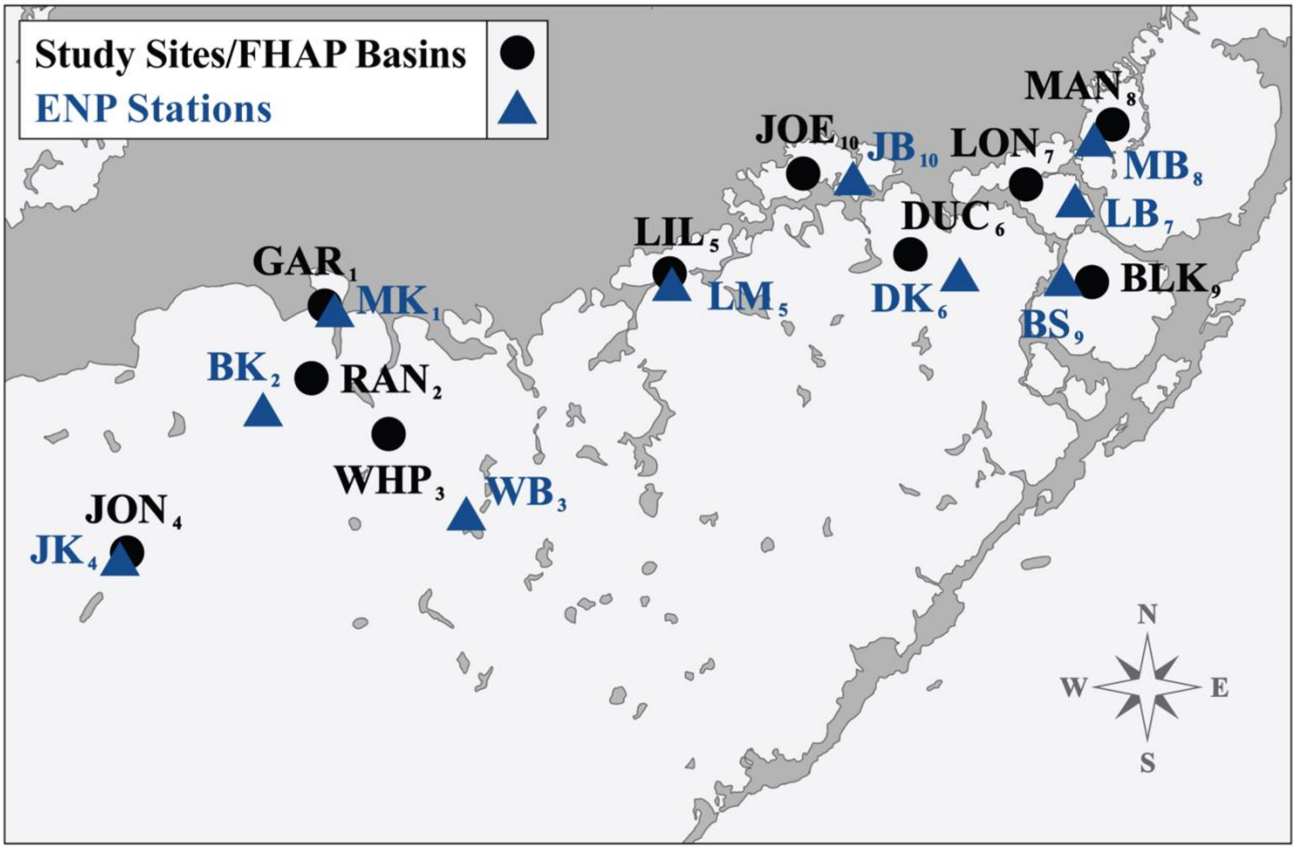

Supplementary Figure 1. Map detailing the locations surveyed for turtlegrass and/or salinity measurements within Florida Bay. The 15 main turtlegrass collection sites associated with this study (black circles) corresponded with permanent transect basins established by the Fisheries Habitat Assessment Program (FHAP) and long-term monitoring stations through the South Florida Water Management District (SFWMD), the latter of which collects water quality data (including salinity) on a monthly basis. A second set of monitored regions (blue triangles) coordinated through the Everglades National Park (ENP) were sampled for daily salinity measurements when ENP locations were considered proximate to our main study sites (subscripts denote location pairings).

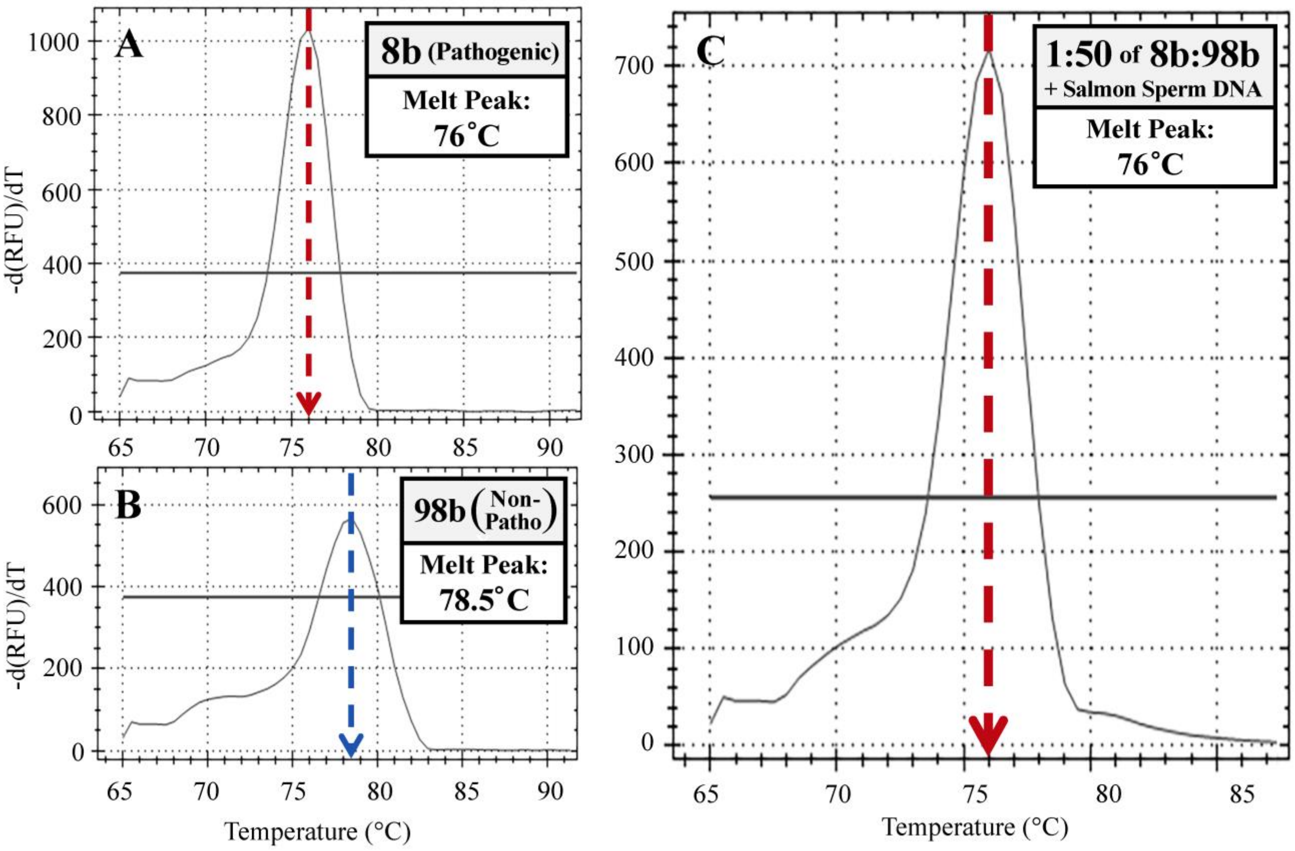

Supplementary Figure 2. Melt peak profiles of *Labyrinthula* sp. isolates used to demonstrate preferential amplification of pathogenic template DNA through qPCR. (A) pathogenic strain 8b, (B) putatively non-pathogenic 98b, (C) mixture of both strains where 98b template DNA outnumbered 8b template DNA 70 to 1, supplemented with background DNA in the form of salmon sperm DNA (Invitrogen™). Distinctive melt curve peak temperatures served as the primary diagnostic feature between the two isolates, particularly when both templates were present (part C). The chosen melt profiles are representative of duplicate replicates performed for each treatment. See Duffin et al. (2020) for detailed isolate information.

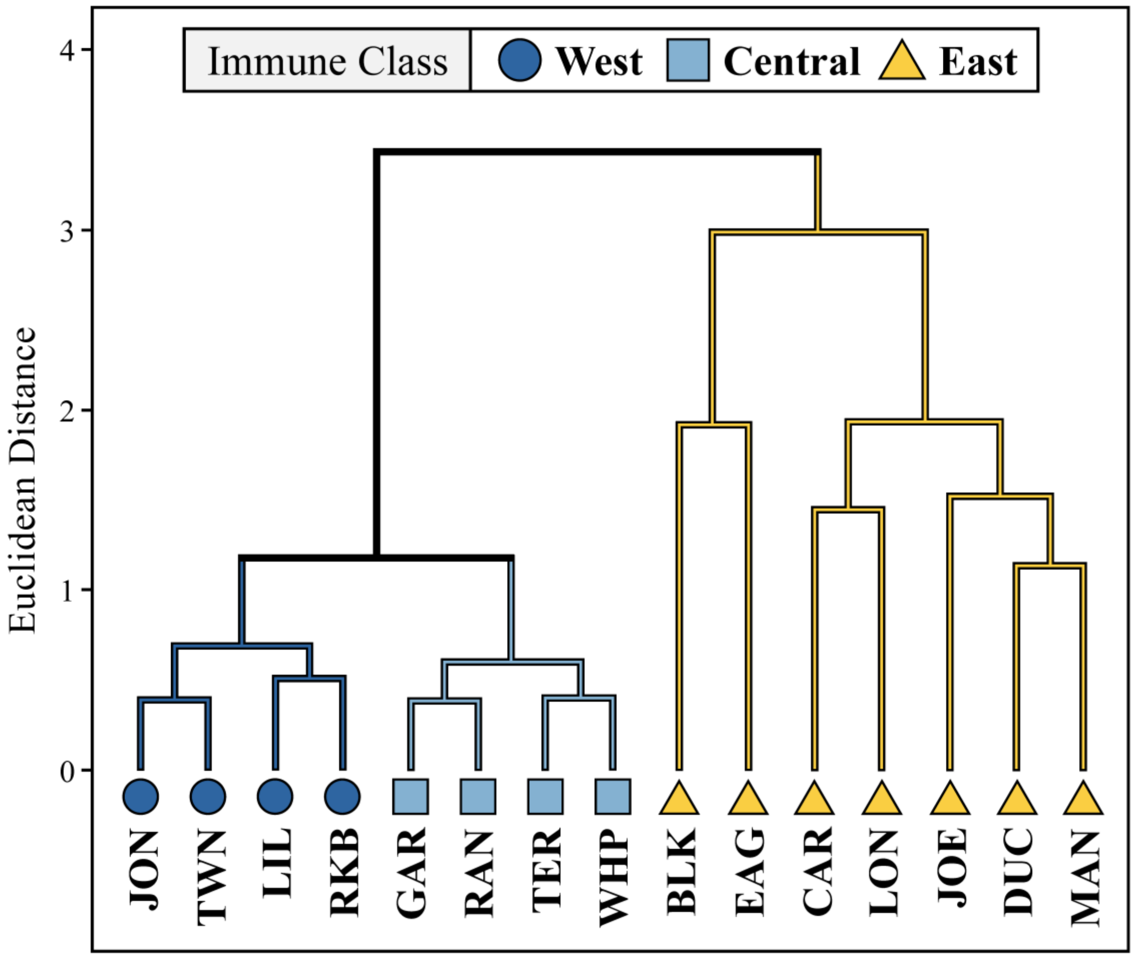

Supplementary Figure 3. CLUSTER dendrogram grouping sites into immune classes based on similarity profiles of average site activity levels at the four biomarkers measured (POX, EXOC, PPO, and LYS activity). Groupings were significant according to a similarity profile routine (SIMPROF, Clarke et al., 2008). Given the congruence these immune classes had with geographical areas of the study region (see Fig. 4), sites were named based on corresponding generalized bay regions (‘West,’ ‘Central,’ and ‘East’) for simplicity.

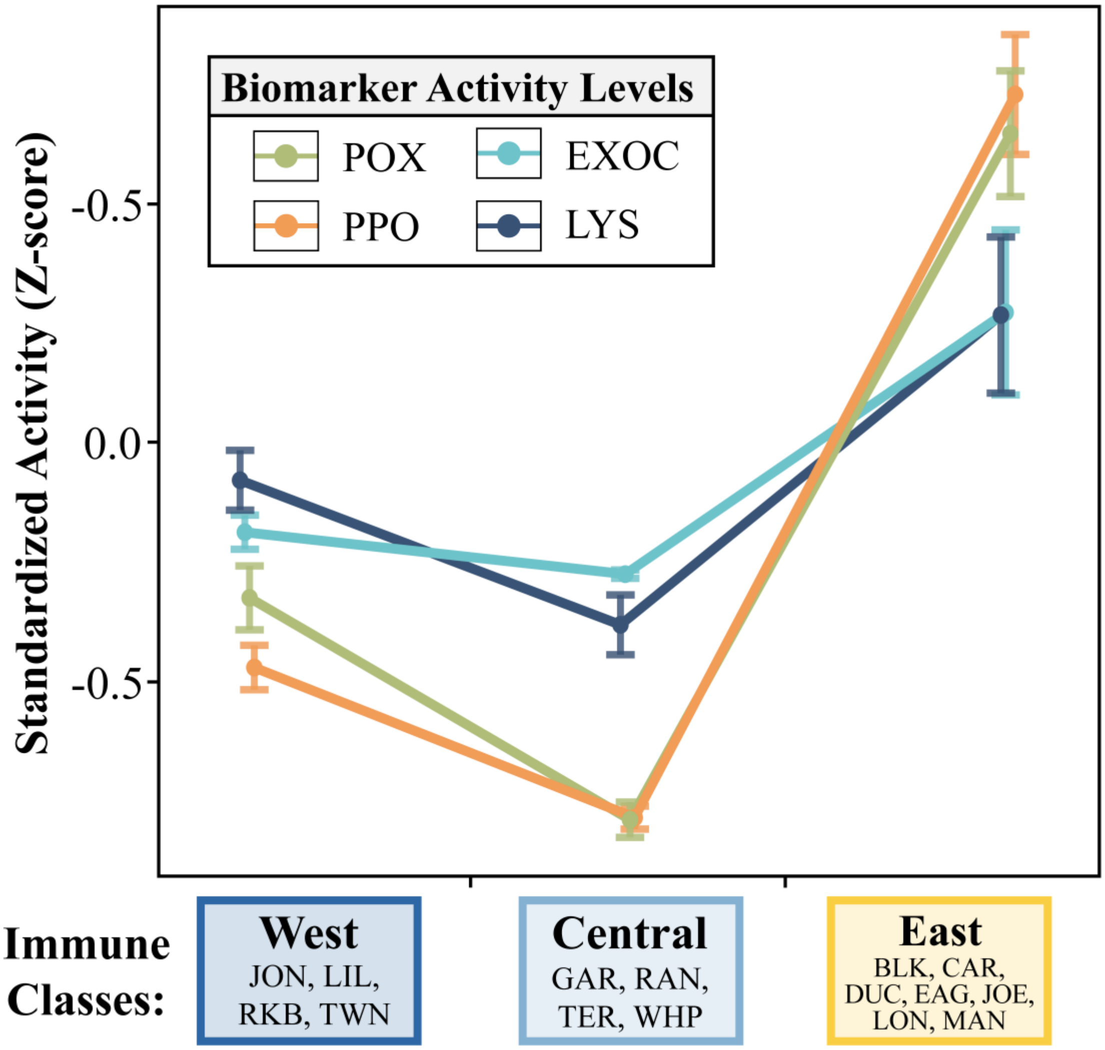

Supplementary Figure 4. Standardized activity levels (Z-scores) at the four biomarkers (POX, EXOC, PPO and LYS) in turtlegrass among three immune classes established through hierarchical clustering. Error bars represent Z-score standard error.
